# On the Influence of Mercurial Preparations upon the Secretion of Bile

**Published:** 1859-11

**Authors:** George Scott


*On the Influence of Mercurial Preparations upon the Secretion of Bile.* By Dr. GEORGE SCOTT. (Beale's Archives of Medicine, No. 3.)

Dr. Scott relates the details of some experiments made on dogs, with the view of ascertaining whether the preparations of mercury really increase the flow of bile, as has hitherto been generally believed. In these experiments the ductus communis choledochus was tied, so as to prevent any bile from reaching the intestine, and the gall-bladder was opened in order to allow all the bile secreted to escape externally. It was then collected in an apparatus constructed for the purpose, and calomel being given at different periods, the quantity of the bile secreted was carefully noticed. In the first place, however, Dr. Scott ascertained the normal amount of bile secreted by a dog's liver in the twenty-four hours. The quantity amounted on an average of three days to 2752·562 grains of fluid bile each day; the average weight of the dog being seventeen pounds, and the average amount of food being 7000 grains, and of drink nineteen ounces, or 8312·5 grains of milk,

In determining the effects of calomel on the secretion of bile, Dr. Scott calculated the average amount of bile secreted in twenty-four hours two days *previously* to the administration of the drug, and then the average amount secreted in twenty-four hours two days *after* the calomel was given. The calomel was given each time after the morning's bile was collected, and therefore the effect of the medicine was upon the bile of the day following that on which it was given. The four experiments performed by Dr. Scott all gave the same rather parodoxical result—namely, that *there was a diminution in the amount of fluid bile and bile-solids secreted after the administration of large doses of calomel.*

Although Dr. Scott considers that it would be rash to venture any decided opinion from the results of four experiments, yet these all point so much to one conclusion that if they should be confirmed by future and more varied trials, they will throw considerable doubt upon the generally received opinion that calomel in large and purgative doses increases the flow of bile. It may be urged, he adds, that although calomel does not increase the secretion of bile in the dog, there is no reason why it may not do so in man; and that even if mercury does not excite the liver to increased secretion in a healthy state of the organ, it may yet do so in some of its diseased conditions. But if the first objection were true, the same could be urged against the results of experiments on the lower animals to ascertain the action of poisons or any articles of the materia medica. With regard to the second objection, nothing analogous occurs in the action of drugs upon other organs; and it seems difficult to suppose that anything which diminishes the flow of bile in a healthy condition of the liver, should increase it in a diseased state of that organ. Whether it be the mere purgative effect of calomel which causes the diminution in the secretion of bile, or some specific action, must be decided by further experiments. It is also a matter for further inquiry, whether small and frequent doses of calomel continued for a length of time, so as to produce the specific action of mercury upon the system, may not really augment the biliary secretion.

